# Commentary: Behavior analysis and behavioral neuroscience

**DOI:** 10.3389/fnhum.2016.00256

**Published:** 2016-06-01

**Authors:** Teresa C. Kolu

**Affiliations:** Cusp EmergenceThornton, CO, USA

**Keywords:** behavior analysis, behavioral neuroscience, single-subject design, operant conditioning, contextual conditioning, hippocampus (Hip)

In this commentary, the author expands on Schlinger's 2015 contribution to the research topic, “Can an Emphasis on the Single Subject Provide Novel Neuroscientific Insights? Where Neuroscience Meets Behavior Analysis.” Whereas Schlinger suggested behavior analysis affords an experimental and theoretical model useful for neuroscientists, this commentary specifies areas of intersection that are currently rich with possibility. Concurrent challenges and opportunities are present for researchers and practitioners interested in better understanding the stimulus control of complex behavioral events or “cognitive processes” through a single-subject informed lens. It is suggested that neuroscience-informed behavior analysis is poised to improve the technology of both measurement and behavior change at the individual level, and that such a relationship could inform research and clinical practices of both behavior analysts and neuroscientists.

How and where can behavioral neuroscientists incorporate single subject design in their existing repertoires? Behavioral neuroscience already employs approximations to incorporating single-subject designs. Czerniawski and Guzowski (2014) used a discrimination ratio to compare in individual rats (whose brains were ultimately compared) behavior at different time points using a “context discrimination” task. Saline injected rats froze in the presence of a contextual stimulus paired previously with footshock, but not in the presence of an unpaired contextual stimulus. The authors modeled immune system activation by injecting lipopolysaccharide, which impaired discriminated behavior and hippocampal CA3 neural networks. This study and others have shown that dorsal hippocampus is specifically recruited in behavioral tasks requiring discrimination but not necessarily contextual fear conditioning alone (Parsons and Otto, 2008, 2010). Consider the implications, in which a small little-understood difference in the laboratory testing environment and experimental design carves or strengthens a path toward decades of similar research: simple contextual fear conditioning manipulations were used for years with little modification to investigate properties of “memory storage and retrieval,” but do not differentially invoke the dorsal CA3 networks of hippocampus as do tasks involving discrimination (Parsons and Otto, 2008). This example illustrates that behavior analysts may be uniquely positioned to support a research community beginning to embrace more complex behavioral tasks. While these studies occurred with nonhuman organisms, improving measurement technology and collaboration is necessary for improving translational research and work with human participants as well. Behavior analysis, suggests Schlinger (2015), may also be useful to neuroscience in investigating events often called “cognitive.” Behavioral scientists can operationalize and examine complex “cognitive” behaviors together with neuroscientists employing single subject designs and behavioral tasks.

In addition to the potential for experimental contributions, collaboration between behavior analysis and neuroscience is poised to inform more individualized clinical treatment and training for health care providers. Behavior analysis informs the evidence-based treatment of psychiatric, genetic, and behavioral disorders and co-occurring challenges. For instance, consequential contingencies often play an important role in hallucinatory behavior (e.g., Layng and Andronis, 1984), which may function as a successful operant. Similarly, echolalia can occur as subvocal, verbal, or challenging behavior used by learners with autism, but can also occur after brain damage in otherwise typically developing individuals. Behavior analysis approaches these and similarly complex events in a person's behavior stream as behavioral, rather than focusing on their cognitive role or description. In turn, the more complex or subtle “behavioral function” of seemingly cognitive processes can be informative to transdisciplinary teams of practitioners. Adding behavioral definition and measurement of “cognitive processes” affords critical data informing treatment decisions including modification of medications and individualized educational, family or behavior plans.

Strengthening connections between human neuroscience and behavior analysis holds theoretical implications for both fields. There is an ethical need to incorporate more effective measurement of the changes experienced by an individual exposed to long-term self-harm or other challenging behavior, drug side effects, and autism treatment. These areas hold a dangerous amount of applied significance, as widespread changes may be taking place in the brains, behavior streams, and behavioral environments of thousands of children prescribed antipsychotics, mood stabilizers, and ADHD medications with insufficiently researched side effects. Further, recent policy driven funding opportunities for children to receive tens of weekly hours of behavior analytic therapy for autism spectrum disorders may pose a new potential intersection between human neuroscientists and behavior analysts. It is consistent with Schlinger's (2015) suggestions to strengthen existing relationships between functional analysis of behavior and behavioral neuroscientific assays of brain function before, during and following the systematic manipulation of various behavioral therapy variables. However, given the increasing need for appropriate behavioral services and better behavioral scientists to collaborate with other fields, a substantial subset of behavior analysts entered the field from other professions, lacking adequate education, experience, and mentorship (including role models who are both scientists and practitioners). Without continued development, these insufficiently trained and supervised analysts cannot adequately respond to other members of the scientific community's mischaracterizations of their field, or confront claims that behavior analysis is simplistic and cannot handle private events. To deepen understanding of behavioral contributions to complex phenomena may require both better dissemination of behavior and nonlinear contingency analysis, and the continued analysis of behavioral environments of scientists.

In closing, this article suggests that consistent with Schlinger's position Schlinger (2015), it is possible and prudent to foster conditions allowing behavior analysis and neuroscience to inform and influence each other. Consider that some behavioral neuroscience preparations with non-human animals require sacrificing subjects, in order to conduct between-subject comparisons of neurochemical changes demanding large sample sizes that obscure potentially important within-subject differences. Interested readers might examine their own definition of experimental control against Sidman's (1960), which refers “to the investigator's ability to manipulate an individual subject's behavior in a precise and reliable fashion…[To] be able to turn some quantitatively consistent aspect of behavior on and off by the manipulation of specifiable variables demonstrates a high order of control” (p. 342). As researchers continue to incorporate single subject designs and operant preparations where appropriate, better control within a single subject may yield important data on the individual's neural changes that accompany and interact with changes in the other, more “accessible” aspects of the behavior stream.

## Author contributions

The author confirms being the sole contributor of this work and approved it for publication.

### Conflict of interest statement

The author declares that the research was conducted in the absence of any commercial or financial relationships that could be construed as a potential conflict of interest.
